# Comparison between Tube Ileostomy and Loop Ileostomy as a Diversion Procedure

**DOI:** 10.5402/2012/547523

**Published:** 2012-12-18

**Authors:** Vijayraj Patil, Abhishek Vijayakumar, M. B. Ajitha, Sharath Kumar L

**Affiliations:** Department of General Surgery, Victoria Hospital, Bangalore Medical College and Research Institute, Bangalore 560002, India

## Abstract

*Aim*. Loop ileostomy has high complication rates and causes much patient inconvenience. This study was performed to compare the outcome of tube versus loop ileostomy in management of ileal perforations. *Patients and Methods*. From July 2008 to July 2011, all patients with ileal perforation on laparotomy where a defunctioning proximal protective loop ileostomy was considered advisable were chosen for study. Patients were randomly assigned to undergo either tube ileostomy or classical loop ileostomy as the diversion procedure. Tube ileostomy was constructed in the fashion of feeding jejunostomy, with postoperative saline irrigation. *Results*. A total of 60 diversion procedures were performed over the period with 30 for each of tube and loop ileostomy. Typhoid and tuberculosis formed the most common etiology for ileal perforation. The complication rate of tube ileostomy was 33%. Main complications related to tube ileostomy were peritubal leak, tube blockage. In patients with loop, overall complications in 53% majority were peristomal skin irritation and wound infection following ileostomy closure. Two patients developed obstruction following ileostomy closure which needed reoperation. *Conclusions*. Tube ileostomy is effective and feasible as a diversion procedure and has reduced morbidity. It can be used as an alternative to loop ileostomy.

## 1. Introduction

Emergency laparotomy for intestinal perforation and obstruction surgeons are faced with difficult decision to perform stoma for fecal diversion; an even more difficult task is explaining the need for stoma to patient. Creation of a diverting stoma has its own set of complications including stomal retraction, prolapse, or necrosis; para-ileostomy infection/abscess and fistula; intestinal obstruction; skin irritation/excoriation; mucosal ulceration; offensive odors; prestomal ileitis; diarrhea; and hemorrhage [1]. The need for frequent change of the costly ileostomy appliance because of the leakage following loss of seal imposes great financial burden, especially in developing countries. A need for second surgery for closure of stoma adds on to financial burden and unnecessary delays due to nonprioritization of stoma closure due to high case volume. The closure of the intestinal stoma is also frequently followed by complications in 17%–27% of patients [2, 3]. 

These complications include fever, wound infection, abdominal septic complications, leak from ileostomy closure, intestinal obstruction, incisional hernia, and death.

We designed this prospective study to assess the feasibility and outcome of proximal catheter ileostomy in place of a defunctioning proximal loop ileostomy in patients treated by primary repair and/or resection-anastomosis of small bowel. The construction of catheter ileostomy was based on the concept of the currently well-accepted catheter jejunostomy. A comparison was made between tube ileostomy and loop ileostomy in terms of complications and outcome.

## 2. Material and Methods

The present study was conducted at Bangalore Medical College and Research Institute (BMCRI), Bangalore, India, from July 2008 to July 2011. Patients who underwent explorative laparotomy for small bowel perforation or obstruction and in whom a decision to perform a proximal diversion stoma on the basis of any of the following intraoperative findings: multiple perforations, edematous and inflamed bowel, adherent loops of bowel, and insecure anastomosis, were chosen for the study. Patients were randomly assigned to undergo either a tube ileostomy or classical loop ileostomy as a diversion procedure.

Patients who died within 5 days of surgery unrelated to anastomotic complication and patients who were lost to followup were excluded from the study.

## 3. Technique of Tube Ileostomy Construction

**Figure 1:**
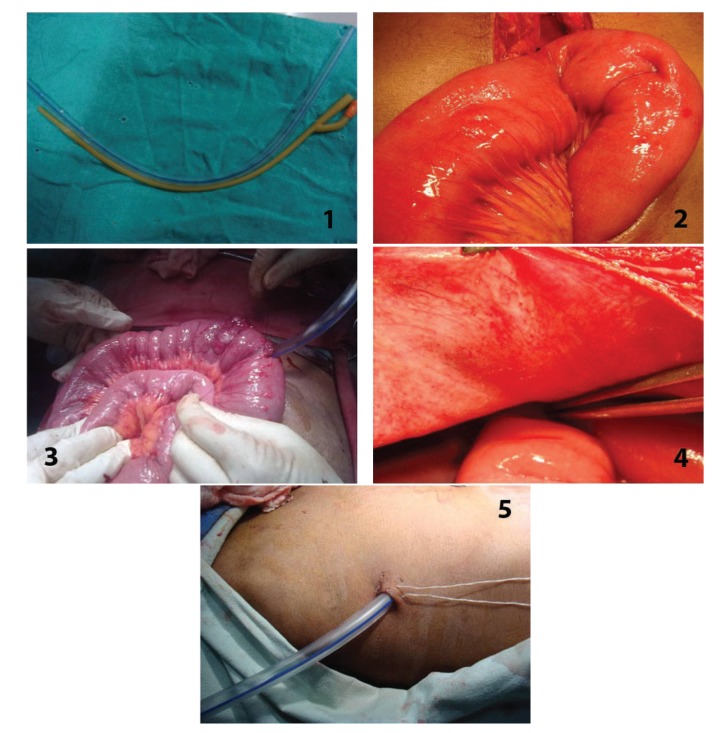
Steps of tube ileostomy. (1) Tube selection abdominal drain or Foley's catheter. (2) Selection of healthy segment of bowel proximal to repair. (3) Tube insertion and anchoring with purse string suture. (4) Fixation to anterior abdominal wall. (5) Fixation of tube to skin.

At laparotomy after dealing with primary pathology and performing necessary procedure, patients underwent either tube ileostomy or open ileostomy. A 28 French abdomen drain tube was brought into peritoneal cavity through stab incision on abdomen wall. Tube was inserted 10 cm proximal in diseased bowel in case of ileal perforation with tube tip directed proximally (Figure 1). Tube was secured to bowel wall by 2-0 polyglactin by purse string suture. Segment of bowel 5 cm proximal and 5 cm distal to the site of insertion of tube was fixed to parietal wall with interrupted 2-0 polyglactin. Tube was fixed to skin with 2-0 Mersilk and connected to drainage bag. Another tube was inserted through flank and placed in pelvis.

 In patients with no evidence of anastomotic, leak tube was clamped after second week and finally removed after third week of surgery to have a controlled fistula in place. Clinical suspicion of leak prompted the tube ileostomy to be maintained till the leak would seal. 

A detailed record of day on which tube ileostomy started functioning, tube drainage, peritubal leak, tube blockade, any feature suggestive of anastomotic leak, or any other complication was maintained. The day when tube was clamped and removed was recorded. Time to closure of the controlled fistula was also noted. All the patients were regularly followed in the outpatient department for any complications.

## 4. Results 

Over a period of three years from July 2008 to July 2011 a total of 60 diversion procedure were performed. Out of the diversion procedures 30 were conventional loop ileostomy and 30 were tube ileostomy as described above.

Patients ranged from 16 to 63 years with mean age 32.6 years. Majority of patients were male (70%).

Preoperative diagnosis was perforation (*n* = 46), obstruction (*n* = 15), and penetrating abdominal injury (*n* = 1). Duration of symptoms ranged from 1 to 5 days. The most common symptoms were pain abdomen (*n* = 53), abdominal distension (*n* = 45), and absolute constipation (*n* = 22). Among presented signs all patients had tachycardia, signs of peritonitis were present in 47 patients with masked liver dullness (*n* = 23), free fluid in abdomen (*n* = 42), and seven patients presented with severe dehydration and shock. 

Erect X-ray showed air under diaphragm in 36 cases, dilated bowel loop in 24 cases, and ground glass appearance in 7 cases. Abdominal ultrasound showed moderate free fluid in 54 of the cases and dilated loops in 28 cases. Widal test was positive in 38 cases with high “*O*” titers in 22 cases, and tuberculin test was positive in 16 cases with 9 cases showing high Adenosine deaminase levels.

During the period of study 13 patients died in immediate postoperative period, unrelated procedure, and related complications, 9 were due to septicemia and multiorgan failure and 2 each as a result of massive pulmonary embolism and myocardial infarction. These cases were excluded from the study.

In majority of patients (64%) tube ileostomy started functioning on first postoperative day while in rest from second day. Tube ileostomy output ranged from 50–700 mL/day with mean of 300 mL. Once a day irrigation was sufficient to keep the tube patent in 25 patients; 5 patients developed tube blockade of whom 4 resolved with thrice daily irrigation of tube with saline. One patient who had persistent blockade and developed signs of peritonitis was reoperated and found to have a kinking of tube and anastomotic leak. Three patients developed peritubal leak which was managed with regular dressing.

The tube ileostomy was removed on postoperative day 21; the drain site managed with daily dressing in whom the wound discharge was minimum. Two patients had increased wound discharge which was managed with application of colostomy bag for 2 weeks which later resolved, and wound closure was achieved. The wound-closure time ranged from 4 to 9 days (mean 7 days). None of the patients required formal closure of the wound. All patients were followed for an average of 6 months and showed no complications. 

In loop ileostomy group the main complication was peristomal skin excoriation (*n* = 4) which required prolonged regular dressing. Two patients developed severe dehydration following high output from stoma and required hospitalization for electrolyte abnormalities and were managed with intravenous fluid. There was one case of early necrosis of stoma and retraction which required operation and stoma revision. Anastomotic leak occurred in two cases one of which required reoperation due to clinical deterioration. The patients underwent ileostomy closure between 2 to 4 months (mean 10 weeks). The main complication following ileostomy closure was wound infection (*n* = 6) which resolved with regular dressing and antibiotics. Two patients developed obstruction following closure one of whom required reoperation. Patients were followed up for a period of 6 months with one patient presenting with obstruction which required reoperation and adhesion release (Tables 1 and 2). 

## 5. Discussion

In the first report of loop ileostomy by Turnbull and Weakley [4] in 1966 it has gained popularity as a method of fecal diversion to protect distal anastomosis. The routine use of loop ileostomy to protect the distal anastomosis is much debated, and the literature supports all arguments both in favour and against. Therefore presently no conclusive evidence or guidelines supports the use or avoidance of such ileostomies. Proponents of routine use argue that though the ileostomy does not prevent leakage, it does however decrease the detrimental effects of a leak [5] with less major leaks and less reoperation rates [6]. 

In cases of intestinal perforation or obstruction with features of peritonitis a defunctioning proximal protective loop ileostomy is considered advisable due to presence of one or more of the following intraoperative findings: insecure repair or anastomosis, multiple perforations, matted bowel loops, and grossly unhealthy bowel due to severe edema and inflammation [7, 8]. 

On the other hand, the routine use of ileostomy adds to the morbidity and mortality besides longer hospital stay and costs. Furthermore because ileostomy closure is not a high priority in this era of stringent financial budgeting, it is often postponed or delayed [9]. During this period stoma-related complications have been reported to range from 9–74% [10–12].

Even minor complications with the ileostomy significantly hamper the quality of life of these patients. Tube ileostomy as an alternative to loop ileostomy is an attempt to protect the distal anastomosis and at the same time decrease the ileostomy complications and totally avoid the morbidity and mortality associated with stoma takedown.

The first reported use of T-tube ileostomy was at Texas Children's Hospital in 1959 for proximal fecal diversion; several investigators have reported successful outcomes following laparotomy with T-tube enterostomy with irrigation in neonates with unresolved uncomplicated meconium ileus unrelieved by contrast enema [13]. In 1981, Lizarralde [14] used lateral tube ileostomy in 23 of 59 children operated upon for typhoid ileal perforation and reported a success rate of 43.5%. Rygl et al. [15] found T-tube ileostomy to be an effective and safe primary repair technique in five extremely low-birth-weight children with localized intestinal damage/perforation.

Use of tube ileostomy in adults is only sparingly reported. With first reported case by Hojo [16] who used tube ileostomy along with total colectomy and ileoanal anastomosis for familial polyposis coli in seven young patients and had successful outcomes in all. He found that the simple tube ileostomy is as effective as the loop ileostomy and recommended the procedure.

In our study we noted complications with tube ileostomy in 33% of patients; this was significantly less than the complication rate after the loop ileostomy of 53%. The peristomal complications like skin breakdown, dermatitis, and erythema with loop ileostomy were 16%, in accordance to that reported in the literature ranging from 3–36% [17]. These problems can be transient but recurrent till ileostomy is closed and usually hamper the proper application of ileostomy appliance making management of ileostomy effluent difficult [18]. In our study local skin problems in tube ileostomy were noted in only three patients due to peritubal leakage; however this problem is transient and easily treatable or preventable by maintaining tube patency by regular flushings. 

 Dehydration requiring frequent hospital admissions is a well-known problem after ileostomy due to high ileostomy output. Reported incidence of dehydration ranges from 2.2–20% [19]. These patients have to be on astringent diet and antidiarrheal medication often [20]. Some recommend such measures only if urinary sodium concentration is low (0–10 meq/L), and delay discharge till effluent is less than 1 L/day. Two of our patients on loop ileostomy presented with dehydration and electrolyte disturbances requiring readmission. 

The other major complications associated with conventional loop ileostomy include prolapsed and retractions. Of these retraction is more worrisome as it results in skin excoriation and incomplete defunctionalization of the distal anastomosis. This problem is reported to occur in up to 15.9% of patients [21]. In our study only one patient developed retraction of loop ileostomy which required reoperation. 

 Incomplete defunctionalization using tube ileostomy occurs if the tube gets blocked and does not drain freely. Tubal blockage was found in five of our patients; it was observed that in patients in whom the tube was placed within 25 cm of ileocecal junction developed block. This could probably be due to increased consistency of feces distally. All tube blocks were managed with saline irrigation thrice daily. In one patient who developed peritonitis and was reoperated there was kinking of the tube which had caused circumferential tear at tube. A resection anastomosis was done followed by a proximal loop ileostomy. 

Early bowel obstruction before loop ileostomy reversal has been reported to be due to adhesions, retraction of loop ileostomy, and herniation of proximal bowel lateral to the ileostomy. Stoma-related obstruction occurred in 6.4% with loop ileostomy in the study reported by Metcalf et al. None of the patients with tube ileostomy developed obstruction. Two patients developed obstruction following closure of loop ileostomy of whom one required reoperation. There have been reports that the risk of obstruction is less if the ileostomy site is resected and anastomosis performed by stapler [22]. However others have not found any significant difference between the various techniques of closure [20].

Leakage from distal anastomotic site with pelvic sepsis despite proximal loop ileostomy is well known. García-Botello et al. [20] reported 10.24% anastomotic leak in series of 127 patients despite proximal loop ileostomy. Four patients (3.15%) had to be reoperated due to generalized peritonitis, worsening clinical signs, or evidence of sepsis despite conservative management. Feinberg et al. reported a 13.6% leak rate in their series of 117 patients [18]. Two patients with loop ileostomy developed anastamostic leak which was initially managed conservatively; one patient required reoperation due to worsening clinical signs.

Other complications have been frequently reported with delay in the ileostomy closure or the takedown itself. Gallstones occur with gallstone pancreatitis especially if the ileostomy closure is delayed [18]. Anastomotic leaks from ileostomy reversal site in up to 8.3% are mentioned in the literature [17, 19]. The incidence of wound infection has been reported from 1.3%–18.3% while incisional hernia occurs in up to 11.9% of patients after ileostomy closure [17]. All these morbidities are avoided with the use of tube ileostomy.

In a study by Rondelli et al. [23] comparing loop ileostomy to percutaneous tube ileostomy in patients undergoing laparoscopic anterior resection for adenocarcinoma, a balloon catheter was used for tube ileostomy and inflation of balloon to achieve complete defunctioning ileostomy. We feel a complete defunctioning ileostomy is not necessary for anastomosis healing and an adequate decrease in flow across the anastomosis using tube ileostomy is enough. Also for successful tube ileostomy care must be taken to place the tube in healthy segment of bowel. Very proximal placement of tube leads to high volume ileostomy output. And when tube is placed in distal ileum a larger lumen tube must be used to prevent tube blockade. Further large scale well-designed, randomized control trials are needed to compare tube ileostomy as an alternative to conventional loop ileostomy as a diversion procedure.

## 6. Conclusion 

Tube ileostomy was found to be an alternate diversion procedure with few complications and was easy to construct and manage as compared to conventional ileostomy. It effectively diverts the bowel contents and avoids the need for a second surgery and its related complications. Further larger randomized studies need to be undertaken before tube ileostomy could be recommended as an alternative to loop ileostomy as a diversion procedure. 

## Figures and Tables

**Table 1:** Characteristics of cases.

Characteristics	Tube ileostomy		Loop ileostomy	
	*N *	%	*N *	%
Age in years				
<20	2	6.6	0	0
20–30	11	36.6	10	33.3
30–40	12	40.0	15	50.0
40–50	4	13.3	3	10.0
>50	1	3.3	2	6.6
Sex				
Male	22	73.3	19	63.3
Female	8	26.6	11	36.6
Etiology				
Typhoid	20	66.6	18	60.0
Tubercular	6	20.0	4	13.3
Traumatic	1	3.3	0	0
Nonspecific	3	10.0	8	26.6

**Table 2:** Complications following tube and loop ileostomy.

	N	%
Tube ileostomy		
Peritubal leak	3	10.0
Tube block	5	16.6
Distal anastomotic leak	1	3.3
Tube migration	1	3.3
Loop ileostomy		
Peristomal skin irritation	4	13.3
Electrolyte imbalance	2	6.6
Necrosis	1	3.3
Retraction	1	3.3
Post ileostomy closure obstruction	2	6.6
Wound infection	6	20.0
